# Efficacy and predictability of Muller’s muscle-conjunctival resection with different tarsectomy lengths for unilateral blepharoptosis treatment

**DOI:** 10.1186/s12886-021-01849-y

**Published:** 2021-02-12

**Authors:** So-Hung Yeh, Shu-Lang Liao, Yi-Hsuan Wei

**Affiliations:** 1 College of Medicine, National Taiwan University, Taipei, Taiwan; 2 Department of Ophthalmology, National Taiwan University Hospital, 12F, No.7, Chung Shan S. Rd., Zhongzheng Dist., Taipei, Taiwan; 3 Department of Ophthalmology, National Taiwan University College of Medicine, Taipei, Taiwan

**Keywords:** Ptosis, Muller’s muscle-conjunctival resection, Tarsectomy, Algorithm, Treatment

## Abstract

**Background:**

To investigate the efficacy and predictability of Muller’s muscle-conjunctival resection (MMCR) with different lengths of tarsectomy for the treatment of unilateral mild-to-moderate blepharoptosis.

**Methods:**

A retrospective study of patients who underwent MMCR with tarsectomy for unilateral mild-to-moderate blepharoptosis between January 2016 and December 2019 was performed. Individuals with adequate photographic documentation and good levator function were included. Data on age, gender, surgical designs, pre-operative and post-operative marginal reflex distance 1 (MRD1) and tarsal platform show (TPS), and complications were retrieved.

**Results:**

Sixty patients underwent 8-mm MMCR with 1- or 2-mm tarsectomy; 53 patients (88.3%) showed postoperative symmetry of MRD1 within 1 mm. The average postoperative improvement in MRD1 was 2.15 ± 0.8 mm. Thirty-two patients received 8-mm MMCR with 1-mm tarsectomy (group 1), and 28 patients underwent 8-mm MMCR with 2-mm tarsectomy (group 2). In group 1, postoperative symmetry rate was 90.6%, and the mean elevation of MRD1 was 1.66 ± 0.6 mm. In group 2, postoperative symmetry rate was 85.7%, and the mean elevation of MRD1 was 2.72 ± 0.6 mm. Both groups showed postoperative symmetry of TPS and significant improvement in eyelid position (*p* < 0.0001). No postoperative complication was noted, and no secondary surgery was needed.

**Conclusions:**

MMCR with tarsectomy was proven to be a safe, rapid, and effective method for patients with mild-to-moderate ptosis. Predictability and symmetry of the outcome were statistically confirmed. We further suggest a 2.1-mm expected MRD1 elevation as a cut point for choosing between 1- or 2-mm tarsectomy.

**Supplementary Information:**

The online version contains supplementary material available at 10.1186/s12886-021-01849-y.

## Background

According to the severity of blepharoptosis and levator muscle function of the patient, there are numerous treatment options that a surgeon can choose for ptosis repair. For patients with mild-to-moderate ptosis and sufficient levator muscle function, Muller’s muscle-conjunctival resection (MMCR) is the procedure of choice. MMCR was first described by Putterman and Urist in 1975 [1], and it has increased in its popularity due to its cosmetic improvement, fast recovery, lack of external scar, and simplicity. MMCR may have several surgical designs [2–4] and variable resection lengths, and some studies have compared the results of MMCR with or without tarsectomy [5–7]. In our previous surgical experience, MMCR alone without tarsectomy did not achieve satisfactory results in our Asian patients. We preferred to perform MMCR with tarsectomy to augment the surgical efficacy. Meanwhile, how much correction we may achieve under different resection lengths is always one of the top issues for surgeons and thus could be found in many studies [8–11]. However, only few studies were designed to explore the relationship between the length of tarsus resected and the change of eyelid position [7].

The purpose of this study was to analyze the surgical results of MMCR with different lengths of tarsectomy. We investigated the relationship among tissue resection length, marginal reflex distance 1 (MRD1), tarsal platform show (TPS), and change in MRD1 in individuals undergoing MMCR surgery with different resection lengths of tarsectomy. We also investigated the symmetry of the outcome, considering that it is an indicator of success for unilateral ptosis surgery. Furthermore, the study aims to determine whether longer tarsus resection length could lead to greater eyelid elevation. This will help us to predict the approximate required tarsus resection length according to the severity of ptosis.

## Methods

This is a retrospective, single-center review that was approved by the Institutional Review Board of National Taiwan University Hospital. The patients received MMCR with different tarsectomy lengths performed by a single surgeon (Y.H.W.) at National Taiwan University Hospital between Jan. 2016 and Dec. 2019. Inclusion criteria included age > 18 years, unilateral mild-to-moderate ptosis, good levator muscle function (> 8 mm), positive 2.5% phenylephrine test, follow-up of at least 6 months. Mild ptosis was defined as 2 mm or less difference of MRD1 between the ptotic and healthy eye and moderate ptosis as 2 to 4 mm difference. All patients included in our study was categorized into acquired ptosis, and all of them were aponeurotic type. Exclusion criteria included any previous eyelid trauma or surgery, or history of systemic disease that could potentially affect eyelid position. Patients with insufficient recorded data were also excluded from this study. All cases received 8-mm MMCR and an additional 1 mm or 2 mm tarsectomy chosen according to the severity of ptosis. Specifically, additional 1 mm tarsectomy was performed for patients with mild ptosis, while additional 2 mm was performed for patients with moderate ptosis.

### Surgical technique

Surgery was performed under local anesthesia in all patients. The upper eyelid skin and superior fornix were infiltrated with lidocaine 2% with 1:100000 epinephrine. The upper eyelid was everted over a Desmarres retractor, and markings for the traction sutures were made with marking pen on the conjunctiva 4 mm from the superior tarsal border.

Three silk traction sutures were placed along the markings centrally, medially, and temporally passing through the conjunctiva and Muller’s muscle. Markings for tarsectomy were made with a marking pen on the tarsus, 1- or 2-mm away from the superior tarsal border. With all three traction sutures pulled simultaneously ventrally towards the ceiling, a Putterman clamp was applied to engage the tissues to be excised including the conjunctiva, Muller’s muscle, and tarsus. A 6-0 prolene continuous suture was passed through the skin to the conjunctiva 1 mm inferior to the clamp border. The suture was weaved 3–4 times through the conjunctiva and tarsus in a horizontal mattress fashion along the clamp border. After the suture exited the skin, it was re-introduced into the skin and weaved back through the conjunctiva and tarsus and exited the skin near the first entry site. The ends were tied externally with an approximate tension, and the clamped tissues were cut free along the clamp border with a #15 blade. After hemostasis with cauterization, pressure patching was applied for 1 day, and the prolene suture was removed 14 days postoperatively. If an overcorrection was noted at the first week, early removal of suture with gentle massage may lower the lid height.

**Fig. 1:**
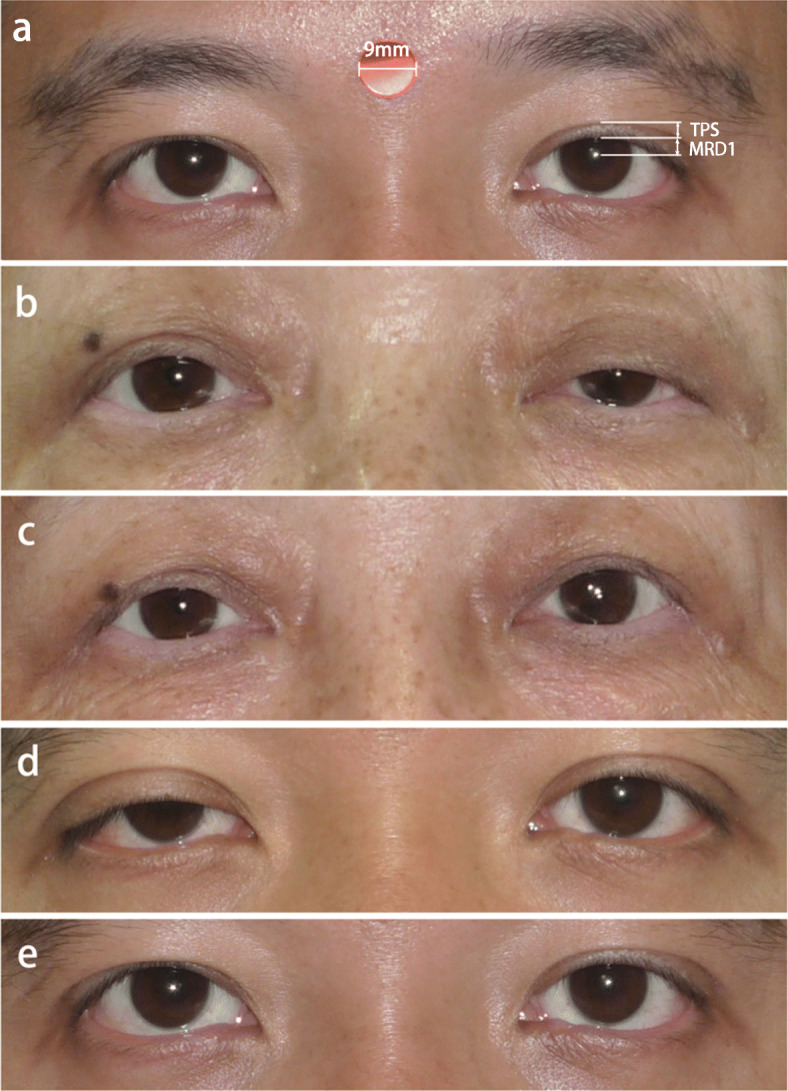
Representative photographs of patients underwent Muller’s muscle-conjunctival resection (MMCR) with tarsectomy. The measurement of marginal reflex distance 1 and tarsal platform show (**a**). Preoperative (**b**) and postoperative (**c**) photographs at 6 month follow up of a 64-year-old female who underwent 8 mm MMCR with 1 mm tarsectomy in her left eye. Preoperative (**d**) and postoperative (**e**) photographs at 6 month follow up of a 37-year-old male who underwent 8 mm MMCR with 2 mm tarsectomy in his right eye

The photos were obtained before the surgery and at 6 months postoperatively with the patients in a sitting position ensuring that the brow was stabilized and the frontalis muscle was not recruited. Before taking the photos, we put a standard red marking dot of 9 mm in diameter on the forehead between the eyebrows as a reference in setting the measurement scale. All photographs of the patients were reviewed in a standard manner and all measurements were performed using IC Measure version 2.0.0.161 (Supplement Fig. 1). MRD1 was measured from the center of the pupil to the lowermost margin of the upper eyelid in the mid-pupillary line, while TPS was measured from the top of the visible tarsal plate to the upper eyelid margin (Fig. 1a). Analyzed data included age, gender, surgical designs, pre-operative and 6-month post-operative MRD1 and TPS, and complications.

Statistical analysis was performed with t-test to detect the statistically significant differences of MRD1 and TPS after the surgery as well as the differences between the two surgical designs. For linear regression and correlation analyses, Pearson’s correlation coefficient was used, while logistic regression was used to detect the factors that will affect the surgical success significantly. Data were analyzed using IBM SPSS Statistics for Mac version 21.0 (IBM Corp. Released 2012. Armonk, NY: IBM Corp.). Charts and diagrams were sketched using GraphPad Prism 8.0.1(GraphPad Software Inc., La Jolla, San Jose, CA, USA). The study was powered for a β error of 0.95 and α of 0.05.

## Result

A total of 60 eyes from 60 patients met the inclusion criteria and were included in the final sample. All cases were diagnosed with acquired mild-to-moderate unilateral upper eyelid ptosis and received unilateral surgery. In this study, 60 cases were divided into two groups after collecting the data retrospectively: 32 patients received 8-mm MMCR with additional 1-mm tarsectomy (group 1); the other 28 patients underwent 8-mm MMCR with additional 2-mm tarsectomy (group 2). The overall sample was 75% (*n* = 45/60) female with a mean age of 54.2 ± 16.5 years, while 51.7% (*n* = 31/60) of ptotic eyes that received MMCR with tarsectomy was in the right eye. In this retrospective review, both MRD1 and TPS were fully documented before and after the surgery (Table 1) in order to investigate the efficacy and symmetry of the procedure. Four patients without visible crease did not have the data for TPS. For all cases, the average of MRD1 elevation was 2.15 ± 0.8 mm. Symmetry was defined as MRD-1 difference within 1 mm between two eyes and was observed in 88.3% of patients. The representative preoperative and postoperative photographs are shown in Fig. 1b-e. No postoperative complication such as entropion, ectropion, or corneal erosion was noted. Eyelid contour may be slightly changed in few patients, but no secondary surgery was needed.

**Table 1:** Overall data and comparison between group 1 (8-mm MMCR with 1-mm tarsectomy) and group 2 (8-mm MMCR with 2-mm tarsectomy)

	All subjects (***N*** = 60) mean ± SD (range)	Group 1 (***N*** = 32) mean ± SD (range)	Group 2 (***N*** = 28) mean ± SD (range)	***P*** value
Age (years)	54.2 ± 16.5 (23–80)	49.0 ± 16.0 (23–77)	60.0 ± 15.3 (28–80)	0.009
Female (%)	75	78.1	71.4	0.55
Preop MRD1 of ptotic eye (mm)	1.23 ± 0.9 (0.53–3.60)	1.58 ± 0.9 (− 0.33–3.60)	0.83 ± 0.8 (− 0.53–2.44)	0.002
Preop MRD1 of healthy eye (mm)	3.21 ± 1.0 (1.30–5.68)	3.10 ± 0.9 (1.56–5.68)	3.34 ± 1.1 (1.30–5.56)	0.327
Postop MRD1 of ptotic eye (mm)	3.38 ± 0.9 (1.71–5.94)	3.24 ± 0.9 (1.81–5.94)	3.54 ± 0.9 (1.71–5.87)	0.194
Postop MRD1 of healthy eye (mm)	3.07 ± 1.0 (1.11–5.94)	3.06 ± 1.1 (1.11–5.94)	3.09 ± 0.9 (1.52–4.95)	0.909
Preop TPS of ptotic eye (mm)	5.36 ± 1.4 (2.25–10.15)	4.88 ± 1.3 (2.25–8.36)	5.91 ± 1.4 (3.48–10.15)	0.007
Preop TPS of healthy eye (mm)	3.20 ± 1.3 (0.56–6.80)	3.12 ± 1.5 (0.56–6.80)	3.29 ± 1.2 (1.05–6.65)	0.639
Postop TPS of ptotic eye (mm)	3.06 ± 1.2 (0.69–7.10)	3.04 ± 0.9 (1.53–5.94)	3.09 ± 1.5 (0.69–7.10)	0.867
Postop TPS of healthy eye (mm)	3.33 ± 1.3 (0.45–6.88)	3.27 ± 1.4 (0.45–6.79)	3.40 ± 1.3 (1.28–6.88)	0.711
Change in MRD1 (mm)	2.15 ± 0.8 (0.46–3.82)	1.66 ± 0.6 (0.46–3.82)	2.72 ± 0.6 (1.54–3.79)	< 0.001
Postop MRD1 difference within 1 mm between two eyes (%)	88.3	90.6	85.7	0.554

For data involving TPS, group 1 and 2 had 30 and 26 subjects respectively

*MRD1* Marginal reflex distance 1, *Preop* Preoperative, *Postop* Postoperative, *TPS* Tarsal platform show

As shown in Table 1, there was a thorough comparison between group 1 and group 2. Preoperatively, there was no significant difference between the two groups in both MRD1 (3.10 vs. 3.34 mm; *p* = 0.327) and TPS (3.12 vs. 3.29 mm; *p* = 0.639) in the healthy eye. As for the ptotic eye, statistically significant difference was noted in both MRD1 (1.58 vs. 0.83 mm; *p* = 0.002) and TPS before surgery. (4.88 vs. 5.91 mm; *p* = 0.007). Postoperatively, however, there was no significant difference between the two groups in MRD1 and TPS, regardless if the eye is healthy or ptotic (Table 1).

**Fig. 2:**
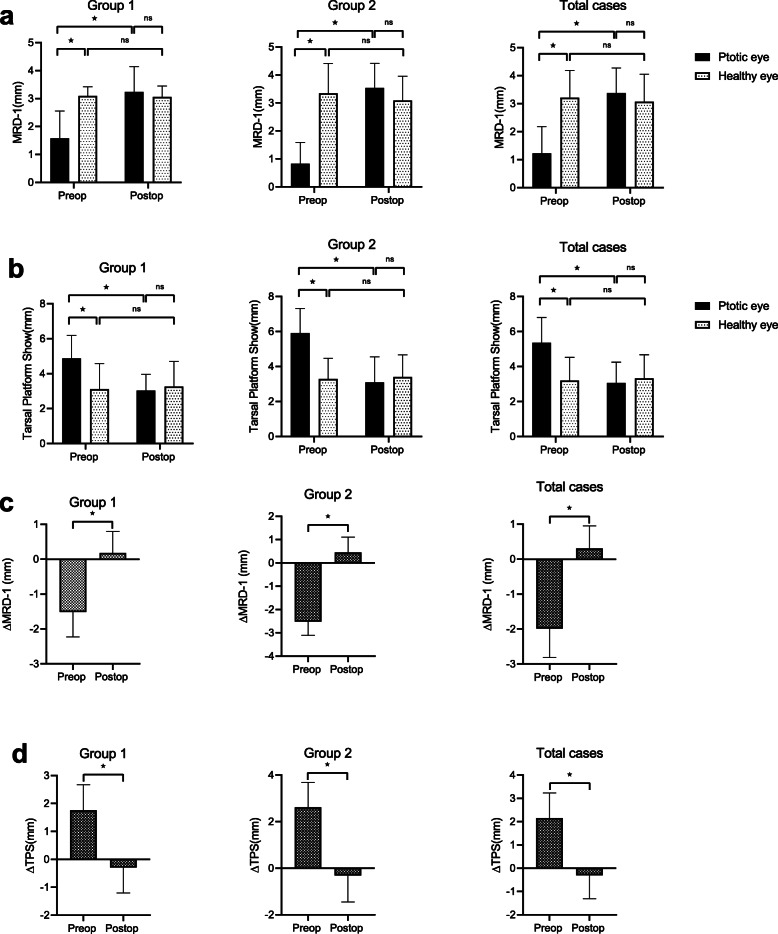
Evaluation of the change and symmetry of marginal reflex distance 1 (MRD1) and tarsal platform show (TPS) before and after Muller’s muscle-conjunctival resection (MMCR) with different lengths of tarsectomy. **a** In total cases and subgroup analysis, there was a significant difference of MRD1 between the two eyes preoperatively, but no significant difference was found postoperatively. For the ptotic eye, there was significant increase of MRD1 postoperatively. **b** In total cases and subgroup analysis, there was a significant difference of TPS between the two eyes preoperatively, but no significant difference was found postoperatively. For the ptotic eye, there was significant decrease of TPS postoperatively. **c** The difference of MRD1 between the two eyes significantly changed postoperatively in total cases and subgroup analysis. **d** The difference of TPS between the two eyes significantly changed postoperatively in total cases and subgroup analysis. *p < 0.05. ∆, difference between the ptotic eye and the healthy fellow eye of each patient

In this study, surgical outcome was examined in group 1, group 2, and all cases. For MRD1, all cases and subgroup analysis showed significant difference preoperatively and no significant difference after surgery (Fig. 2a), while same results were observed in TPS (Fig. 2b). There was no significant difference before and after the operation in the healthy eye (Fig. 2a, b), while the results of the ptotic eye revealed statistically significant improvement in both MRD1 and TPS after surgery (Fig. 2a, b).

Comparing the differences of MRD1 and TPS between the ptotic eye and the healthy eye (∆), all cases and subgroup analysis showed statistically significant difference before and after the operation (Fig. 2c, d). Postoperatively, the mean of MRD1 differences between the two eyes (∆) were less than 0.5 mm in all cases and subgroup analysis (0.3053, 0.1775, 0.4514, mm for all cases, group 1, and group 2 respectively).

**Fig. 3:**
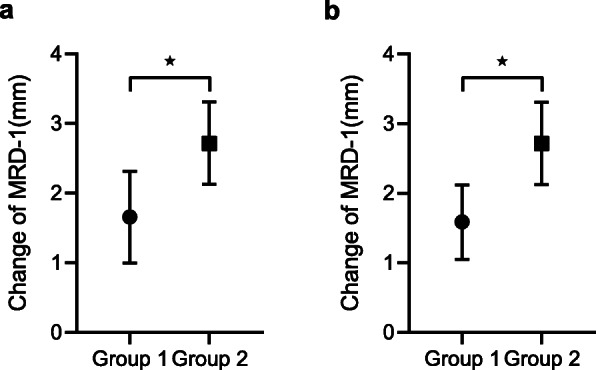
The comparison of the mean change of marginal reflex distance 1 (MRD1) between two surgical groups. **a** The mean change of MRD1 was 1.66 ± 0.66 mm in group 1 and 2.72 ± 0.59 mm in group 2 (*p* < 0.0001). **b** After excluding the outlier data which was out of the range of two standard deviations, the mean change of MRD1 was 1.59 ± 0.53 mm in group 1 and 2.72 ± 0.59 mm in group 2 (*p* < 0.0001)

Post-MMCR improvement in eyelid positions was statistically significant between groups 1 and 2. The mean for group 1 was 1.66 ± 0.66 mm, and 2.72 ± 0.59 mm for group 2 (Fig. 3a). Intuitively, the smaller the overlap between the data of two groups is, the easier for us to set a cut point for surgeons to decide how much tarsus should be resected. Therefore, we excluded the outlier data which was outside the range of two standard deviations (one patient in group 1 excluded). The mean change in MRD1 would then be 1.59 ± 0.53 mm and 2.72 ± 0.59 mm before and after the adjustment respectively (Fig. 3b). Interestingly, the ability of elevating eyelid position between group 1 and 2 showed significant difference and the amount of MRD1 change for both groups was more concentrated, indicating a better predictability of surgical outcome.

## Discussion

In this retrospective review, we examined patients with unilateral mild-to-moderate ptosis undergoing MMCR with two different tarsus resection lengths. For unilateral ptosis surgery, the symmetry of the outcome was especially important. From an aesthetic point of view, the symmetry of MRD1 and TPS were both pivotal. This study aimed to highlight the role of tarsal platform show (TPS) in optimizing the aesthetic outcome of MMCR for unilateral mild-to-moderate ptosis. Compared to external approach such as levator muscle resection which was adjustable during procedure, it is difficult to perform graded adjustment of lid height during MMCR procedure. This makes the predictability and symmetry of the outcome even more important. As we can see in the results, the postoperative symmetry of the surgery was satisfactory when the length of tarsectomy was well-adjusted. The rate of symmetry in this study (88.3%) is comparable with those in other reports that used different techniques and algorithms [10, 12–17].

The internal approach for the correction of blepharoptosis was first mentioned in 1961 by Fasanella and Servat [18]. MMCR was designed by Putterman and Urist soon after [1], suggesting resection length ranged from 8 to 9 mm. The resection amount depends on the response to phenylephrine testing. Since then, MMCR has come with a lot of modified surgical designs [2–4]. Later on, Perry et al. defined a modified algorithm which included tarsectomy into his procedure [7]. They recommended a 9-mm resection of Muller’s muscle and conjunctiva with additional tarsectomy which contributed a 1:1 elevation to eyelid position. A recent study also found that MMCR with tarsectomy could be a safe and effective procedure for treating congenital ptosis [19]. It is worth noting that previous studies had revealed a 4:1 ratio of Muller’s muscle resection length to eyelid elevation [17, 20]. However, according to our clinical observations, this 4:1 algorithm could not be applied properly in most of our Asian patients. This may be due to the greater volume and weight of preseptal fat in the Asian population. Thus, we applied additional tarsectomy to achieve better clinical outcome.

The exact mechanism by which MMCR works for ptosis remains to be elucidated [5]. As for the results of our study, the mechanism and efficacy of this surgical design can be discussed from two different perspectives: MMCR and tarsectomy. Regarding MMCR, the mechanism may be related to plication or scarring of the posterior lamella. Zauberman et al. believed that resecting more Muller muscle did not associate with a higher MRD1 elevation [11]. As for tarsectomy, it was reasonable to assume that each 1 mm of tarsus resected could lead to an approximate 1-mm MRD1 elevation since the tarsus serves as a skeleton for eyelid tissue rather than a contractile tissue. Our data showed that mean changes in MRD1 for 8-mm MMCR with 1-mm tarsectomy and 2-mm tarsectomy were 1.66 and 2.72 mm respectively. This result revealed that an extra 1 mm tarsus resected was equivalent to 1.06 mm of change in MRD1 which was compatible to our conjecture. In conclusion, the mechanism for MMCR with tarsectomy to correct lid position include not only shortening of the posterior lamella [21] but also plication of the levator aponeurosis.

Reducing variability and improving predictability have always been important issues for a surgical design. However, as mentioned above, MMCR surgery does not appear to have a purely mechanical mechanism, causing difficulty in titrating Muller muscle resection length to predict outcomes. On the other hand, tarsus works as a scaffold for eyelid tissue, indicating a potential possibility to control outcome by adjusting tarsus resection length under a 1:1 ratio.

Our results may have implications for understanding the predictability of this procedure. As shown in Fig. 3, analysis after adjustment revealed concentrated distributions for both groups and more importantly, there was no overlapping of value ranges between the two groups. These results suggested that 1- and 2-mm tarsectomies could lead to significantly different ranges of eyelid elevation, making it possible for surgeons to decide the amount of tarsectomy according to the target value. It would be reasonable to set a cut point at approximately 2.1 mm between the two groups. If the patient has a preoperative difference of MRD1 greater than 2.1 mm between his two eyes, MMCR with at least 2-mm tarsectomy would be recommended. On the contrary, if the patient has a difference of MRD1 less than 2.1 mm between his two eyes, MMCR with 1-mm tarsectomy would be the treatment of choice to achieve a symmetrical outcome. By and large, the underlying condition of the patient, levator muscle function, and comprehensive consideration of the surgeon should as well be important factors for the final decision making.

In our study, we used 9 mm reference markers to set the measurement scale, which makes our data even more accurate and reliable. However, limitations of this study include its retrospective nature of data analysis, lack of a masked observer, and the limited sample size. In addition, we did not include severe cases. If we do so, we may have more than two different tarsus resection lengths, making it even more likely to clarify the efficacy of tarsectomy. Further investigation would include an expanded case series with different tarsus resection lengths to further demonstrate its efficacy and potential predictability, hoping to build a comprehensive algorithm for tarsectomy length and expected MRD1 elevation.

## Conclusion

This study provides simplified preoperative evaluation and straightforward surgical design, allowing surgeons to conveniently evaluate and treat patients with unilateral mild-to-moderate blepharoptosis. Our results were statistically satisfactory with most patients showing notable improvement and good symmetry of MRD1 and TPS. From a practical perspective, we suggested a 2.1-mm MRD1 elevation as a cut point for choosing between 1- and 2-mm tarsectomy. In conclusion, MMCR with tarsectomy is a safe, effective, and predictable surgical method for the correction of unilateral mild-to-moderate ptosis.

## Supplementary Information

**Additional file 1: Supplement Fig. 1.** All photographs were measured by IC Measure version 2.0.0.161. Noted that there was a 9-mm reference marker to set the measurement scale.

## Data Availability

The datasets used and/or analysed during the current study are available from the corresponding author on reasonable request.

## Abbreviations

MMCR: Muller’s muscle-conjunctival resection
MRD1: Marginal reflex distance 1
TPS: Tarsal platform show
